# Long-term Outcomes of Transcatheter Arterial Chemoembolization Combined With Radiofrequency Ablation as an Initial Treatment for Early-Stage Hepatocellular Carcinoma

**DOI:** 10.1001/jamanetworkopen.2021.26992

**Published:** 2021-09-27

**Authors:** Yao Jun Zhang, Min Shan Chen, Yong Chen, Wan Yee Lau, Zhenwei Peng

**Affiliations:** 1 Department of Hepatobiliary Oncology, Cancer Center, Sun Yat-sen University, Guangzhou, China; 2 State Key Laboratory of Oncology in Southern China, Guangzhou, China; 3 Department of Radiation Oncology, First Affiliated Hospital, Sun Yat-sen University, Guangzhou, China; 4 Faculty of Medicine, Chinese University of Hong Kong, Prince of Wales Hospital, Shatin, New Territories, Hong Kong SAR, China; 5 Institute of Precision Medicine, First Affiliated Hospital, Sun Yat-sen University, Guangzhou, China

## Abstract

**Importance:**

The long-term outcomes of transcatheter arterial chemoembolization (TACE) combined with radiofrequency ablation (RFA) are not determined.

**Objective:**

To report the long-term outcomes of TACE-RFA.

**Design, Setting, and Participants:**

This cohort study analyzed long-term follow-up data from a phase 3 randomized clinical trial of adults with early HCC conducted from October 2006 to June 2009. Participants were randomly assigned to the TACE-RFA group or the RFA group in a 1:1 ratio and followed up approximately 6 years after the trial was closed. Data analysis was performed March 2020.

**Exposure:**

In the TACE-RFA group, TACE was performed first, and RFA was done 2 weeks later.

**Main Outcomes and Measures:**

Overall survival (OS) and recurrence-free survival (RFS).

**Results:**

Of 189 patients who were included (mean [SD] age, 54.3 [12.0] years; 146 [77.2%] men), 94 and 95 patients were assigned to the TACE-RFA group and RFA group, respectively, with their baseline characteristics well matched. Three patients in each group were lost to follow-up. The 5-year and 7-year OS rates for the TACE-RFA group vs the RFA group were 52.0% and 36.4% vs 43.2% and 19.4%, respectively (hazard ratio [HR], 0.55; 95% CI, 0.39-0.78; *P* = .001). The 5-year and 7-year RFS rates for the TACE-RFA group vs the RFA group were 41.4% and 34.5% vs 27.4% and 18.1%, respectively (HR, 0.66; 95% CI, 0.49-0.89; *P* = .007). On subgroup analysis comparing patients who had tumors larger than 3 cm with those who had tumors 3 cm or smaller, the OS and RFS survival rates in the TACE-RFA group (HR, 3.20; 95% CI, 1.91-5.35, *P* < .001) were significantly better than those in the RFA group (HR, 2.03; 95% CI, 1.30-3.17; *P* = .002).

**Conclusions and Relevance:**

In this cohort study, combined RFA and TACE was associated with better survival than RFA alone on long-term follow-up. Patients with tumors 3 cm or smaller did not benefit as well as patients with tumors larger than 3 cm from the combined treatment.

## Key Points

**Question:**

Is transcatheter arterial chemoembolization combined with radiofrequency ablation associated with better long-term survival outcomes than radiofrequency ablation alone among patients with early hepatocellular carcinoma?

**Findings:**

In this cohort study of 189 patients with early hepatocellular carcinoma, patients treated with transcatheter arterial chemoembolization and radiofrequency ablation experienced better survival outcomes than those who received radiofrequency ablation alone.

**Meaning:**

These results suggest that transcatheter arterial chemoembolization with radiofrequency ablation may be a better first-line treatment than radiofrequency ablation alone for patients with early hepatocellular carcinoma.

## Teaser

This cohort study using long-term follow-up data from a clinical trial examines outcomes associated with adding transcatheter arterial chemoembolization to radiofrequency ablation for treatment of early hepatocellular carcinoma.

## Introduction

Radiofrequency ablation (RFA) kills tumor cells by generating heat using a high-frequency alternating current. The heat leads to coagulative necrosis in tumorous and adjacent liver parenchymal cells. RFA is currently considered as a curative treatment in selected patients with hepatocellular carcinoma (HCC). During the past few decades, studies comparing therapeutic effectiveness of RFA with surgical resection have been conducted.^1,2,3^ RFA is now established as a first-line therapy for very early and early HCC according to the Barcelona Clinic Liver Cancer treatment strategy^4^ because of its excellent effectiveness, minimal invasiveness, and convenient availability. However, as tumor size increases, HCC recurrence after RFA becomes more common than after liver resection.^5^ Large tumors are known to associate with higher incidences of adjacent microvascular invasion and micrometastases, important contributing factors to recurrence after HCC treatment.^6^ Thus, RFA can only be used to treat small tumors as the extent of RFA in destroying the tumor and its adjacent surrounding tissues is limited. The other reason for the high recurrence rate after RFA is that heating can result in an increase in intratumoral pressure, which can displace and diffuse tumor cells through iatrogenic arterioportal fistula or intratumoral shunt created around the ablated zone.^7,8,9^

Thus, a major challenge of RFA is in prevention of HCC recurrence. Transarterial chemoembolization (TACE) before RFA has been investigated in several studies to reduce HCC recurrence rates and to prolong patient survival after RFA. The synergistic cytotoxic effects of combining TACE with RFA in treating small and medium-sized HCC have been demonstrated in several studies,^10,11,12^ most of which are retrospective studies. In a previously reported randomized clinical trial conducted by our team,^12^ TACE-RFA was found to be safe, with superior survival outcomes compared with RFA alone in patients with HCC of less than 7 cm in diameter. This study aimed to analyze the long-term survival outcomes and treatment safety of TACE-RFA vs RFA alone based on long-term follow-up of patients who were recruited into our previously reported randomized clinical trial (RCT).^12^

## Methods

### Study Design and Patients

This follow-up study was approved by the ethics committee of the Cancer Center of the Sun Yat-sen University, and it conformed to the standards of the Declaration of Helsinki.^13^ All patients gave written informed consent to this long-term prospective follow-up study, which was based on a randomized phase 3 trial that has been previously reported (ClinicalTrials.gov identifier: NCT00554905; conducted from October 2006 to June 2009, data analysis performed in February 2012).^12^ The report of this study followed the Strengthening the Reporting of Observational Studies in Epidemiology (STROBE) reporting guideline for cohort studies.

Patients were enrolled into 2 groups: the TACE-RFA group and RFA alone group. The main eligibility criteria were patients with: (1) age 18 to 75 years; (2) a solitary HCC 7 cm or smaller in diameter or multiple (3 or fewer) HCC tumors each 3 cm in diameter or smaller; (3) no radiologic evidence of invasion into major portal or hepatic venous branches; (4) no extrahepatic metastases; (5) an Eastern Cooperative Oncology Group performance status of 0; and (6) Child-Pugh class A or B.^6^ The key exclusion criteria were severe coagulation disorders and evidence of hepatic decompensation.

### Treatment Protocols

In the TACE-RFA group, TACE was performed first, and RFA was done within 2 weeks of TACE. In the RFA group, RFA alone was performed. Details of the treatment protocol can be found in our previous study.^12^

### Follow-up and Treatments for Recurrence

Four weeks after RFA treatment, a 3-phase intravenous enhanced contrast computed tomography (CT) was routinely performed to assess the extent of the treated areas. If residual viable tumor tissues were found, an additional session of RFA was given. Treatment failure was defined as any nodule enhancement shown on a 3-phase enhanced-contrast CT carried out 4 weeks after the additional session of RFA. For these patients, TACE was recommended. Patients were followed-up once every 3 months for the first 2 years, once every 6 months for 2 to 5 years, and once every year after 5 years. At each follow-up visit, ultrasound and blood tests including serum liver function tests and α-fetoprotein (AFP) were carried out. Chest radiography was performed once every 6 months for the first 5 years, and thereafter once every year.

When HCC recurrence was detected, the patients were treated with RFA, TACE, systemic therapy including chemotherapy, targeted therapy and immunotherapy, or conservative treatment depending on the site of recurrence, liver function, and general condition of patients.

### Statistical Analysis

Statistical analyses were performed using the SPSS version 19.0 statistical software (IBM). Comparisons between the 2 groups were done using the *t* test for continuous data and the χ^2^ test for categorical data. Survival curves were constructed by the Kaplan-Meier method and compared by the Cox proportional hazards model with stratification by tumor size and tumor number. Overall survival (OS) was defined as the time between randomization and death from all causes; OS from recurrence to death was also calculated. Recurrence-free survival (RFS) was defined as the time between randomization and recurrence or death from all causes. Adverse effects were assessed using the National Cancer Institute Common Toxicity Criteria grading version 4.0.^14^ The relative prognostic significance of the variables in predicting OS and RFS rates was assessed using multivariate Cox proportional hazards regression analysis. Independent prognostic factors were identified through stepwise selection in a Cox regression model. Added variables that were significantly related to survival on univariate analysis (*P* < .05) were subsequently included in the multivariate Cox model. Results were given as median values with standard deviations. All statistical tests were 2-sided, and a significant difference was considered when *P* < .05. The last follow-up was December 31, 2019. Analysis of the long-term data was performed in March 2020.

## Results

### Patients

**Figure 1.**
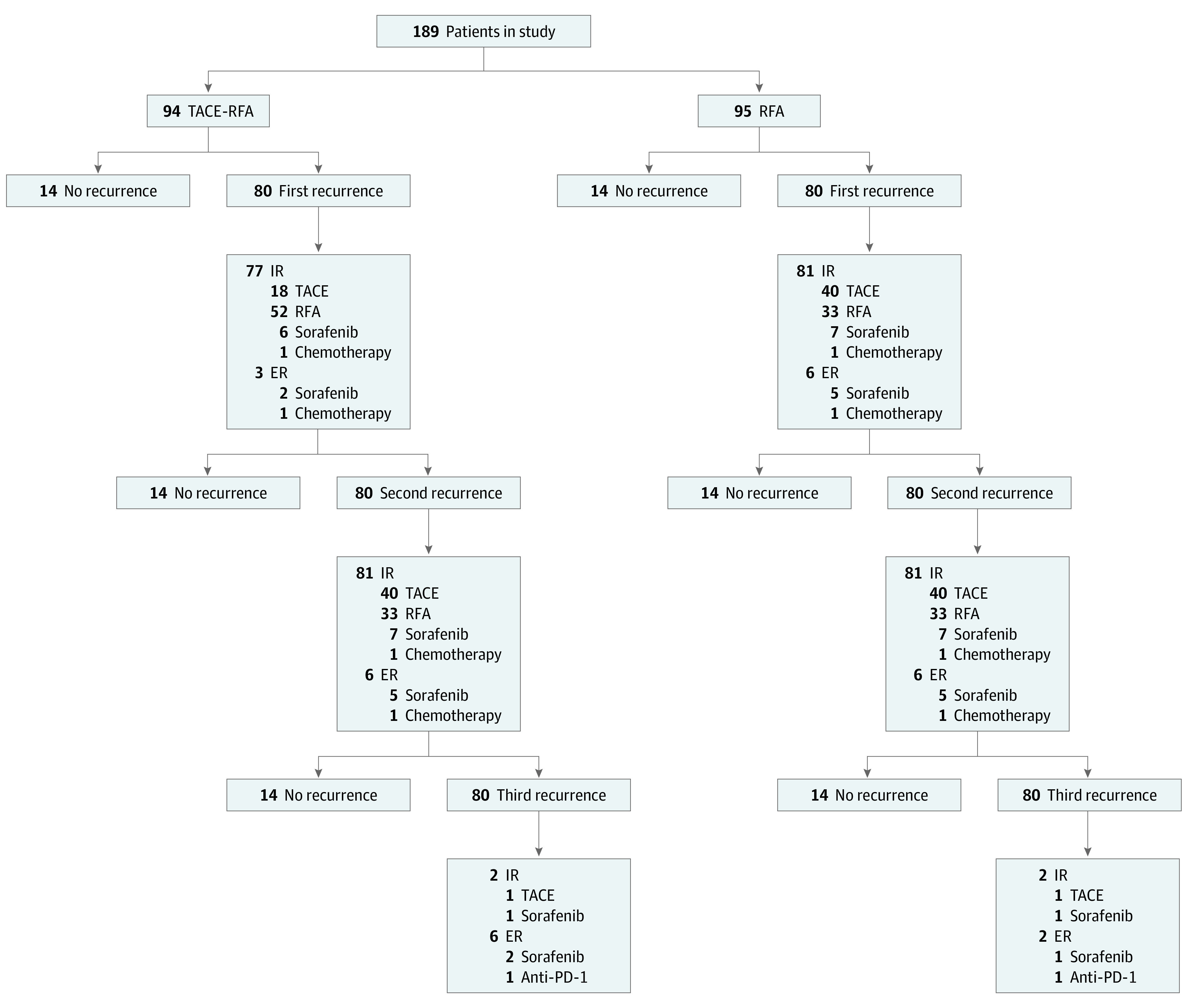
Flowchart of the Study ER indicates extrahepatic recurrence; IR, intrahepatic recurrence; PD, progressive disease; RFA, radiofrequency ablation; TACE, transcatheter arterial chemoembolization.

The flowchart of the study is shown in Figure 1. From October 2006 to June 2009, 189 patients met the inclusion criteria and were randomly assigned to the TACE-RFA group (94 patients) or the RFA alone group (95 patients). One patient in each group withdrew consent from the trial after randomization but agreed to be followed-up by us according to the scheduled protocol. Three patients in each group were lost to long-term follow-up. Baseline demographics and clinical characteristics were well matched between the 2 groups (Table 1). There were 146 men (77.2%) in the study, with 75 patients (79.8%) in the TACE-RFA group and 71 patients (74.7%) in the RFA group.

**Table 1.** Baseline Characteristics of the Patients

Characteristics	Patients, No. (%)	
	TACE-RFA (n = 94)	RFA (n = 95)
Age, mean (SD), y	53.3 (11.0)	55.3 (13.3)
Sex		
Men	75 (80)	71 (75)
Women	19 (20)	24 (25)
HBsAg		
Positive	85 (90)	83 (87)
Negative	9 (10)	12 (13)
HCV-Ab		
Positive	6 (6)	6 (6)
Negative	88 (94)	89 (94)
AFP, ng/mL		
<200	61 (65)	64 (67)
200-400	9 (10)	11 (12)
>400	24 (26)	20 (21)
No. of tumors		
1	62 (66)	67 (71)
2	21 (22)	18 (19)
3	11 (12)	10 (11)
Size of main tumor, mean (SD), cm	3.47 (1.44)	3.39 (1.35)
Size range of tumor size, cm		
≤3	43 (46)	46 (48)
>3	51 (54)	49 (52)
GGT, mean (SD), U/L	65.7 (30.3)	68.4 (28.9)
AST, mean (SD), U/L	44.0 (29.3)	42.0 (24.1)
ALT, mean (SD), U/L	35.0 (10.3)	33.6 (9.7)
TBIL, mean (SD), mg/dL	0.79 (0.17)	0.81 (0.19)
PLT, mean (SD), × 10^3^/μL	121 (65)	118 (64)
PT, mean (SD), %	77.6 (9.4)	76.8 (10.1)
ALB, g/dL		
<35	1.3 (1.4)	1.5 (1.6)
≥35	8.1 (8.6)	8.0 (8.4)
ICGR15		
<10%	72 (77)	74 (78)
10%-19.9%	18 (19)	17 (18)
≥20%	4 (4)	4 (4)
Child-Pugh class		
A	90 (96)	90 (95)
B	4 (4)	5 (5)
Ascites (yes/no)		
Yes	6 (6)	7 (7)
No	88 (94)	88 (93)

Abbreviations: AFP, α-fetoprotein; ALB, albumin; ALT, alanine aminotransferase; AST, aspartate aminotransferase; GGT, Ƴ-glutamyltransferase; HBsAg, hepatitis B surface antigen; HCV-Ab, hepatitis C virus antibody; ICGR15, indocyanine green retention rate in 15 minutes; PLT, platelet count; PT, prothrombin time; RFA, radiofrequency ablation; TBIL, total bilirubin; TACE, transcatheter arterial chemoembolization.

SI conversion factor: To convert AFP to μg/L, multiply by 1.0; GGT to μkat/L, multiply by 0.0167; AST to μkat/L, multiply by 0.0167; ALT to μkat/L, multiply by 0.0167; TBIL to μmol/L, multiply by 17.104; PLT to × 10^9^/L, multiply by 1.0; ALB to g/L, multiply by 10.

### Technical Success of RFA, Recurrence, and Treatment

In the TACE-RFA group, technical success of RFA was achieved after 1 session in 91 patients and 2 sessions in 3 patients. In the RFA group, 88 and 4 patients achieved technical success after 1 and 2 sessions of RFA, respectively. The remaining 3 patients from the RFA group with a tumor larger than 3 cm still had viable tumors after 2 sessions of RFA—they received TACE.

The median (SD) follow-up was 56 (36.6; range, 6-152) months and 50 (34.0; range, 6-149) months for the TACE-RFA or RFA groups, respectively (*P* = .04). On follow-up, 80 of 94 patients (85%) in the TACE-RFA group and 87 of 95 patients (92%) in the RFA group developed first HCC recurrence. (Baseline characteristics of these patients with first tumor recurrence are presented in eTable 1 in the Supplement; details of treatments of the first tumor recurrence are presented in Figure 1 and eTable 2 in the Supplement). Significantly more patients underwent treatment aiming at cure using RFA in the TACE-RFA group than the RFA group (28 of 52 [54%] vs 33 of 54 [61%] patients, *P* < .001; eTable 2 in the Supplement). Of these patients, 36 of 52 patients (69%) in the TACE-RFA group and 27 of 33 patients (82%) in the RFA group developed second tumor recurrence. There were no significant differences between the TACE-RFA and RFA groups in the types and treatments for second tumor recurrence (eTable 3 and 4 in the Supplement).

### Survival

At the time of censoring, 63 patients in the TACE-RFA group and 80 patients in the RFA group had died. The main cause of death was tumor progression (114 of 143 deaths [79.7%]). Tumor progression leading to death occurred in 45 patients (47.9%) in the TACE-RFA group and 69 patients (72.6%) in the RFA group. Other causes of death included liver failure and variceal bleeding (eTable 5 in the Supplement).

**Figure 2.**
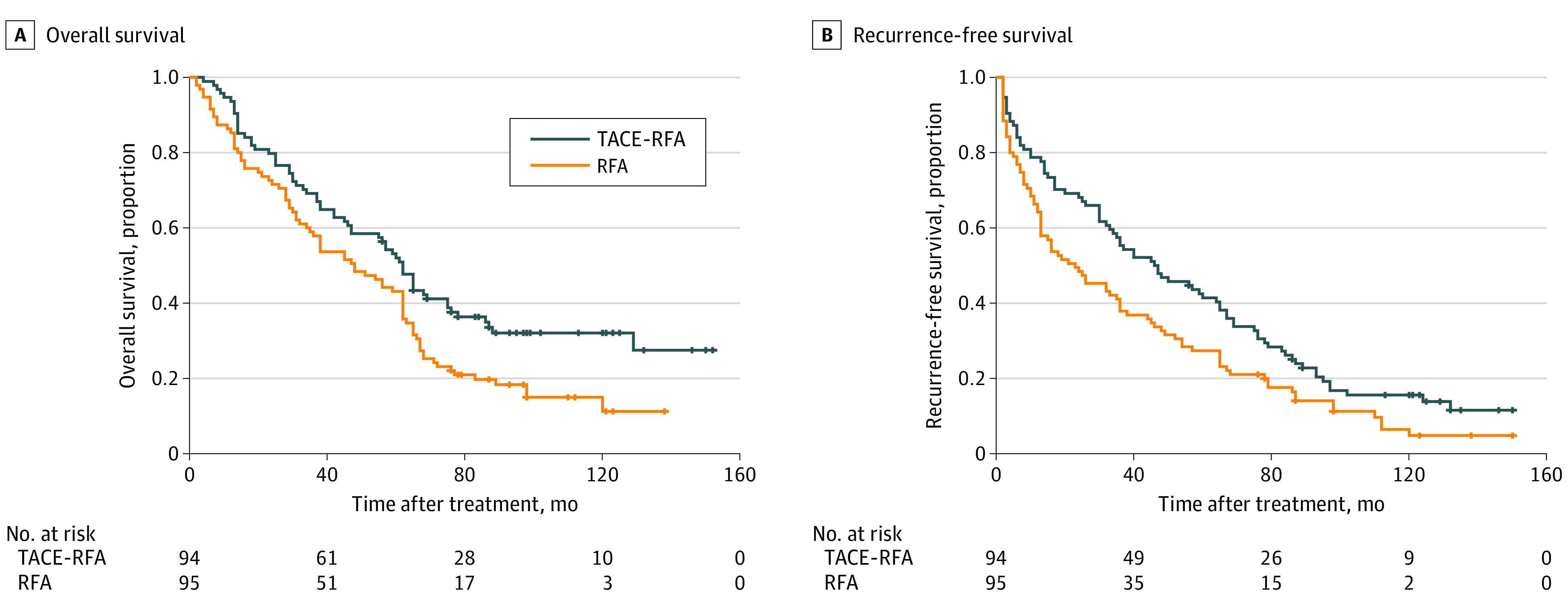
Overall and Recurrence-Free Survival Curves for the Transcatheter Arterial Chemoembolization (TACE) Plus Radiofrequency Ablation (RFA) and RFA Groups

Median (SD) overall survival was 62.0 (3.6) months for the TACE-RFA group (95% CI, 55.0-69.0 months) and 48.0 (4.0) months for the RFA group (95% CI, 33.3-62.7 months). The overall survival rates at 1, 3, 5, and 7 years for the TACE-RFA group vs the RFA group were 94.9%, 69.1%, 52.0%, and 36.4% vs 85.4%, 57.9%, 43.2%, and 19.4%, respectively. The TACE-RFA group had significantly better overall survival outcomes than the RFA group (hazard ratio [HR], 0.55; 95% CI, 0.39-0.78; *P* = .001; Figure 2).

Median (SD) recurrence-free survival was 46.0 (8.8) months for the TACE-RFA group (95% CI, 27.0-65.0 months) and 25.0 (7.6) months for the RFA group (95% CI, 10.5-34.5 months). The recurrence-free survival rates at 1, 3, 5, and 7 years for the TACE-RFA group vs the RFA group were 78.7%, 54.3%, 41.4%, and 34.5% vs 64.2%, 37.9%, 27.4%, and 18.1%, respectively. The TACE-RFA group showed significantly better recurrence-free survival outcomes than the RFA group (HR, 0.66; 95% CI, 0.49-0.89; *P* = .007; Figure 2).

From the time of first tumor recurrence, the 1-year, 3-year, 5-year, and 7-year overall survival rates for patients with recurrence in TACE-RFA group vs those in the RFA group were 49.3%, 12.3%, 5.5% and 3.4%, vs 36.8%, 14.9%, 2.3% and 1.1%, respectively (*P* = .24, eFigure in the Supplement). Median (SD) OS rates were 12.0 (1.8) months for the TACE-RFA group (95% CI, 8.5-15.5 months) and 8.0 (2.3) months for the RFA group (95% CI, 3.52-12.5 months).

### Multivariate and Subgroup Analyses

Multivariate analysis demonstrated that RFA alone (HR, 1.78; 95% CI, 1.26-2.51; *P* = .001) and tumor size (HR, 1.64; 95% CI, 1.14-2.35; *P* = .007) were risk factors of overall survival. Treatment allocation (HR, 1.50; 95% CI, 1.11-2.03; *P* = .009) was the only significant prognostic factor of recurrence-free survival (Table 2).

**Table 2.** Univariate and Multivariate Analyses of Factors in Overall Survival and Recurrence-Free Survival After Treatment

Factors	Overall survival			Recurrence-free survival		
	*P* value for univariate	Multivariate		*P* value for univariate	Multivariate	
		HR (95% CI)	*P* value		HR (95% CI)	*P* value
Tumor size (≤3/>3), cm	<.001	1.64 (1.14-2.35)	.007	.01	1.24 (0.89-1.74)	.20
No. of tumors (≤1/>1)	.02	1.27 (0.90-1.79)	.18	.03	0.98 (0.72-1.35)	.91
AFP (≤400/>400), ng/mL	.01	0.95 (0.82-1.10)	.51	.02	0.97 (0.85-1.11)	.63
Treatment allocation (RFA vs TACE-RFA)	<.001	1.78 (1.26-2.51)	.001	.001	1.497 (1.105-2.029)	.009

Abbreviations: AFP, α-fetoprotein; HR, hazard ratio; RFA, radiofrequency ablation; TACE, transcatheter arterial chemoembolization.

SI conversion factor: To convert AFP to μg/L, multiply by 1.0.

**Figure 3.**
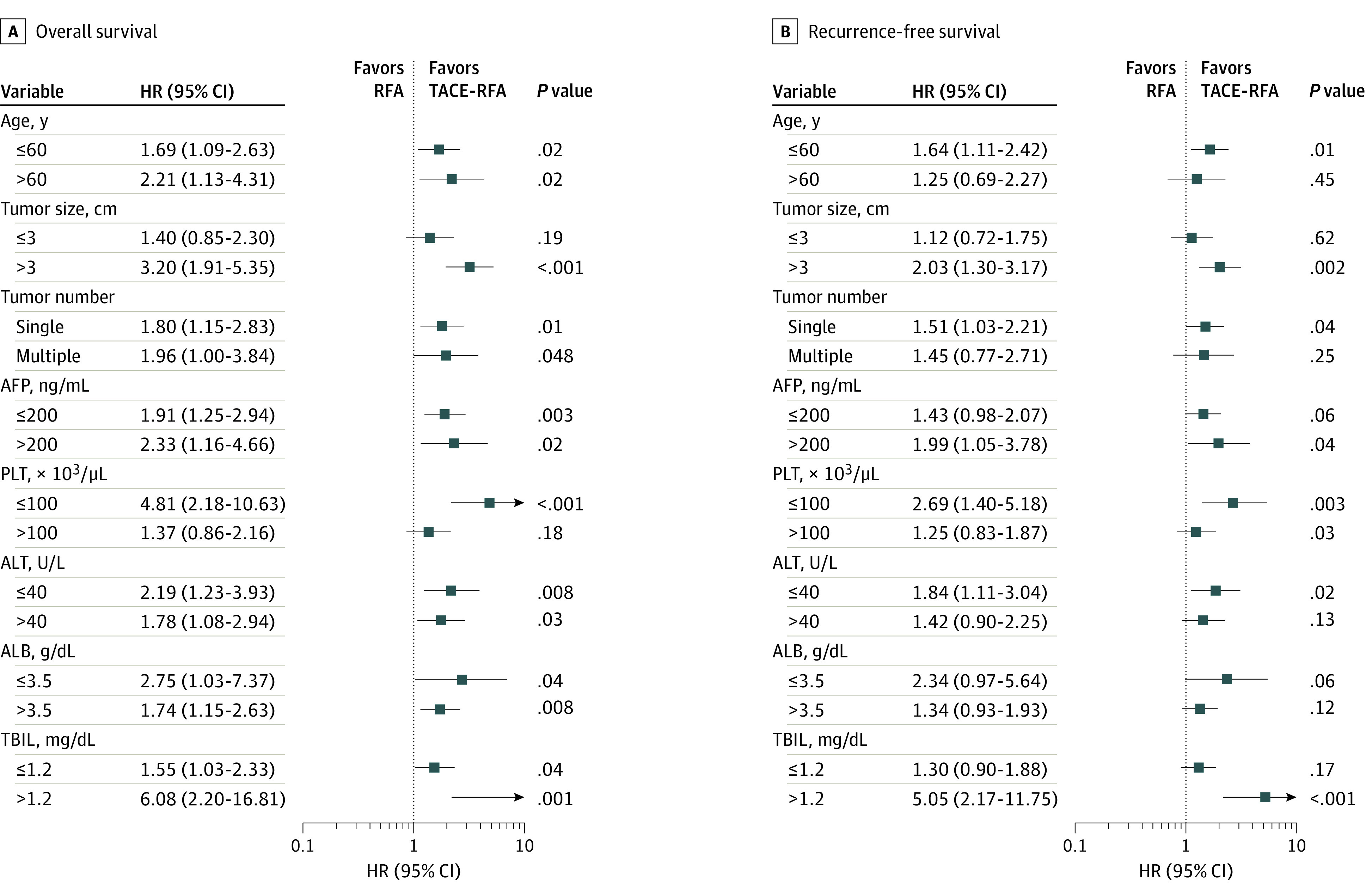
Subgroup Analyses of Overall and Recurrence-Free Survival for Comparing Transcatheter Arterial Chemoembolization (TACE) Plus Radiofrequency Ablation (RFA) and RFA Groups AFP indicates α-fetoprotein; PLT, platelet count; ALT, alanine aminotransferase; ALB, albumin; TBIL, total bilirubin. To convert AFP to μg/L, multiply by 1.0; PLT to × 10^9^/L, multiply by 1.0; ALT to μkat/L, multiply by 0.0167; ALB to g/L, multiply by 10; TBIL to μmol/L, multiply by 17.104.

On subgroup analysis, RFA alone was associated with significantly worse overall survival when compared with TACE-RFA in patients of any age, with any tumor number, AFP level, alanine aminotransferase level, albumin level, total bilirubin level, tumor diameter of greater than 3 cm (HR, 3.20; 95% CI, 1.91-5.35; *P* < .001), and platelet count of less than 100 × 10^9^ (HR, 4.81; 95% CI, 2.18-10.63; *P* < .001) (Figure 3). RFA alone was associated with significantly worse recurrence-free survival when compared with TACE-RFA in patients of age less than 60 years (HR, 1.64; 95% CI, 1.11-2.42; *P* = .01), with tumor diameter greater than 3 cm (HR, 2.03; 95% CI, 1.30-3.17; *P* = .002), single tumor (HR, 1.51; 95% CI, 1.03-2.21; *P* = .04), AFP greater than 200 ng/mL (HR, 1.99; 95% CI, 1.05-3.78; *P* = .04) (to convert AFP to micrograms per liter, multiply by 1.0), platelet count less than 100 × 10^3^/μL (HR, 2.69; 95% CI, 1.40-5.18; *P* = .003) (to convert platelet count to × 10^9^ per liter, multiply by 1.0), alanine aminotransferase less than 40 U/L (HR, 1.84; 95% CI, 1.11-3.04; *P* = .017) (to convert alanine aminotransferase to microkatals per liter, multiply by 0.0167), and total bilirubin greater than 1.2 mg/dL (HR, 5.05; 95% CI, 2.17-11.75; *P* < .001) (to convert total bilirubin to micromoles per liter, multiply by 17.104) (Figure 3).

### Complications

There were no treatment-related deaths. Details of treatment complications can be found in our previous report (and eTable 6 in the Supplement).^12^ Treatment complications, including ascites, pleural effusion, bile duct stenosis, and gastric hemorrhage were also comparable between the 2 groups.

## Discussion

The present study found that TACE-RFA was associated with significantly better overall survival than RFA alone on long-term follow-up. When compared with RFA alone, TACE-RFA was associated with a 45% reduction in risk of death and 34% reduction in risk of HCC recurrence. Patients who received TACE-RFA achieved a median overall survival greater than 5 years and a median recurrence-free survival of approximately 4 years, indicating that this combination treatment was associated with good tumor control for early HCC and should be recommended over RFA alone in clinical practice.

Two factors are known to influence treatment results of RFA and can lead to incomplete ablation and early HCC recurrence. The first factor is the heat sink effect, which is caused by the presence of large blood vessels adjacent to the tumor. The other factor is undetected micrometastases or microvascular invasion from the main tumor.^15,16,17,18^ Because TACE before RFA decreases blood flow to the tumor from the hepatic artery, it reduces the heat sink effect, with resultant increases in the extent of an RFA-induced coagulation zone. Furthermore, TACE decreases the chance of incomplete ablation, a known negative prognostic factor of overall survival after RFA,^19,20,21^ by eliminating any existing micrometastases or microvascular invasion through embolization and chemotherapy. TACE, by inducing ischemia and inflammation in the treated tumor and its surrounding tissues, also increases the extent of the zone of ablative necrosis by RFA.^22,23^ Some studies suggested increased intratumoral pressure generated by the heat from RFA can lead to development of aggressive intrasegmental HCC recurrence.^7,24^ TACE reduces intratumoral pressure by embolizing the arterial supply to the tumor and decreasing the number of tumor cells by chemoembolization, further explaining why patients can benefit from the combination treatment.

On subgroup analysis, this study showed that most patients with a tumor size of up to 7 cm experienced benefit associated with the combined TACE and RFA treatment when excluding HCC patients with a tumor size of less than 3 cm in diameter. Many HCC treatment guidelines have recommended RFA as a first-line therapy for early-stage HCC with a solitary small HCC. However, whether the tumor size should be smaller than 2 cm or 3 cm is still controversial because of evidence that the risk of incomplete tumor necrosis treated by RFA increases with tumor size.^4,25,26,27^

There are several reasons why an early and solitary HCC of less than 3 cm in diameter should be treated by RFA alone. First, several studies have demonstrated that RFA alone can achieve complete ablation in almost 100% of patients with a tumor of less than 3 cm in diameters.^25,26,27^ In our study, the complete ablation rate was 100%. Second, with advances in technology, RFA alone can achieve a necrotic zone of up to 5 cm in diameter. Thus, RFA alone can achieve a complete necrotic marginal zone of 1 cm for a tumor of less than 3 cm in diameter. Third, a previous report showed that there were significantly fewer intratumoral portal tracts in early HCC compared with advanced HCC, but in early HCC there were also significantly more intratumoral arterioles.^28^ Small HCCs, which have a predominant arterial blood supply, are still partly supplied by portal veins that cannot be embolized by TACE, thus explaining why combined RFA and TACE in treating early HCC was not as effective in this study as more advanced HCC. Fourth, there are potential increases in patient discomfort, prolongation of hospital stay, and increases in treatment costs using the combined TACE and RFA treatment.

In this study, significantly more patients in the TACE-RFA group received treatments aiming at a cure for their cancer than the RFA alone group for first tumor recurrence. On analyzing whether further treatments for tumor recurrence affected overall survival outcomes, the median overall survival was 18.1 months in the TACE-RFA group, which was comparable with those patients in the RFA group. Thus, the survival benefit mainly came from the initial treatment. Multivariate analysis also showed that when compared with RFA alone, TACE-RFA significantly reduced the risk of death. TACE-RFA should be recommended over RFA alone as a first-line treatment for patients with early HCC.

Most HCC in this study were resectable, and some HCC met the criteria of liver transplantation. Before enrolling these patients into the RCT, these patients were informed that liver resection and transplantation were other treatment options. However, these patients refused these treatments because of their concerns on cost and/or complications after surgery or liver transplantation. TACE-RFA can be the initial treatment when patients do not accept alternative treatments of liver resection or transplantation.

### Limitations

There are several limitations to this study. First, the number of patients in this study is relatively small. Second, this is a single center study. Third, the results may not be applicable to patients with HCC in other countries with different demographics and etiologies of HCC. Fourth, the initial study was not double-masked because of the nature of treatment and the associated adverse effects of these treatments. However, masking was maintained in evaluating tumor responses by radiologists and in analyzing data by statisticians. Fifth, no patients who received TACE alone were included in the study. TACE has been recommended to be the first-line treatment for intermediate HCC^4^ and early HCC.^29,30^ However, the complete response rates varied from 42.6% to 71.6%, which were significantly less than that by using RFA alone.^30^ Moreover, the recurrence-free survival after TACE was also significantly lower than that after RFA alone treatment.^30^ In this study, TACE was not used to treat early HCC as it was considered not to be a treatment aiming at cure. The strengths of this study are the long-term follow-up, with only 6 patients who were lost to follow-up (3.2%). Such results are solid and reliable.

## Conclusions

In conclusion, combined RFA and TACE was associated with significantly better survival than RFA alone on long-term follow-up. Patients with tumors 3 cm or smaller did not benefit as much as patients with tumors larger than 3 cm using the combined treatment.

## Notes

Supplement. **eTable 1.** Baseline Characteristics of the Patients With First Recurrence After Treatment **eTable 2.** Treatments of First Recurrence **eTable 3.** Types of Second Recurrence **eTable 4.** Treatments of Second Recurrence **eTable 5.** Causes of Death During Follow-up **eTable 6.** Complications After TACE-RFA and RFA **eFigure.** Overall Survival Curves for Patients With Recurrence in the Transcatheter Arterial Chemoembolization (TACE) Plus Radiofrequency Ablation (RFA) and RFA Groups
