# Errors in Figure 1

**DOI:** 10.1001/jamanetworkopen.2021.48091

**Published:** 2022-01-24

In the Original Investigation titled “Long-term Outcomes of Transcatheter Arterial Chemoembolization Combined With Radiofrequency Ablation as an Initial Treatment for Early-Stage Hepatocellular Carcinoma,”^1^ published September 27, 2021, there were errors in the number of patients included at several stages of the flow diagram in Figure 1. This article has been corrected.^1^
