# Supplementary material for: Long-term Outcomes of Transcatheter Arterial Chemoembolization Combined With Radiofrequency Ablation as an Initial Treatment for Early-Stage Hepatocellular Carcinoma
Source: JAMA Netw Open. 2021 Sep 27;4(9):e2126992. doi: 10.1001/jamanetworkopen.2021.26992 (PMC8477266; doi:10.1001/jamanetworkopen.2021.26992)
Supplement: Supplement. — eTable 1. Baseline Characteristics of the Patients With First Recurrence After Treatment eTable 2. Treatments of First Recurrence eTable 3. Types of Second Recurrence eTable 4. Treatments of Second Recurrence eTable 5. Causes of Death During Follow-up eTable 6. Complications After TACE-RFA and RFA eFigure. Overall Survival Curves for Patients With Recurrence in the Transcatheter Arterial Chemoembolization (TACE) Plus Radiofrequency Ablation (RFA) and RFA Groups [file jamanetwopen-e2126992-s001.pdf]

Supplemental Online Content

Zhang YJ, Chen MS, Chen Y, Lau WY, Peng Z. Long-term results of transcatheter arterial chemoembolization combined with radiofrequency ablation as an initial treatment for early-stage hepatocellular carcinoma. *JAMA Netw Open.* 2021;4(9):e2126992. doi:10.1001/jamanetworkopen.2021.26992

eTable 1. Baseline Characteristics of the Patients With First Recurrence After Treatment

eTable 2. Treatments of First Recurrence

eTable 3. Types of Second Recurrence

eTable 4. Treatments of Second Recurrence

eTable 5. Causes of Death During Follow-up

eTable 6. Complications After TACE-RFA and RFA

eFigure. Overall Survival Curves for Patients With Recurrence in the Transcatheter Arterial Chemoembolization (TACE) Plus Radiofrequency Ablation (RFA) and RFA Groups

This supplemental material has been provided by the authors to give readers additional information about their work.

© 2021 Zhang YJ et al. *JAMA Network Open.*

eTable 1. Baseline characteristics of the patients with first recurrence after treatment.

Characteristics	TACE-RFA(n=80)	RFA(n=87)	*P* value
Age, Mean (SD), y	56.5(12.1)	55.4(12.7)	0.542
Male/female	66/14	67/20	0.444
HBsAg (+/-)	75/5	80/7	0.768
HCV-Ab (+/-)	6/74	5/82	0.759
AFP, ng/ml			0.936
<200	37	41	
200-400	17	16	
>400	26	30	
Tumor number			0.037
1	49	38	
2	20	31	
3	11	18	
Size of main tumor, Mean (SD),cm	3.21(1.35)	3.02(1.26)	0.675
Size range of tumor size, cm			0.742
⩽3	55	57	
>3	25	30	
GGT, Mean (SD), u/L	127.3 (96.9)	135.1(98.5)	0.103
AST, Mean (SD),u/L	36.0(21.0)	34.1(23.2)	0.123
ALT, Mean (SD),u/L	30.1(24.6)	29.8(26.3)	0.865
TBIL, Mean (SD),umol/L	16.3(9.1)	17.2(7.9)	0.186

© 2021 Zhang YJ et al. *JAMA Network Open*.

PLT, Mean (SD),10E^9/L	135(46)	130(21)	0.087
PT, Mean (SD), %	85.2(8.9)	79.9(11.2)	0.112
ALB,g/L			0.847
<35	15	18	
≥35	65	69	
Child-Pugh class			0.856
A	60	67	
B	20	20	
Ascites (yes/no)	17/63	17/70	0.849
Types of first recurrence(Intrahepatic/ Extrahepatic/ Both)	77/3/3	81/6/5	0.482

© 2021 Zhang YJ et al. *JAMA Network Open.*

eTable 2. Treatments of first recurrence.

Treatment of first recurrence	TACE-RFA(n=80)	RFA(n=87)	*P* value
TACE	18	40	0.002
RFA	52	33	<0.001
Sorafenib	8	12	0.484
Chemotherapy	2	2	0.999

© 2021 Zhang YJ et al. *JAMA Network Open.*

eTable 3. Tyes of second recurrence.

Types of second recurrence	TACE-RFA(n=36)	RFA(n=27)
Intrahepatic	31	23
Extrahepatic	5	4
Both	5	4

© 2021 Zhang YJ et al. *JAMA Network Open.*

eTable 4. Treatments of second recurrence.

Treatment of second recurrence	TACE-RFA(n=36)	RFA(n=27)
TACE	17	12
RFA	6	4
Sorafenib	9	9
Chemotherapy	2	1
Radiotherapy	2	1

© 2021 Zhang YJ et al. *JAMA Network Open.*

eTable 5. Causes of death during follow-up.

Causes of death	TACE-RFA(n=94)	RFA(n=95)	*P* value
Tumor progression	45	69	0.001
Liver failure with stable disease	14	8	0.181
Variceal bleeding	3	2	0.682
Other	1	1	0.999

Abbreviations: TACE, transcatheter arterial chemoembolization; RFA, radiofrequency ablation.

© 2021 Zhang YJ et al. *JAMA Network Open.*

eTable 6. Complications after TACE-RFA and RFA.

Complications	TACE-RFA(n=94)	RFA(n=95)
Pain	57	51
Grade 1	44	42
Grade 2	11	8
Grade 3	2	1
Fever (temperature> 38.5°C)	33	26
Vomiting	40	29
Grade 2	36	28
Grade 3	4	1
Ascites	5	4
Pleural effusion	3	2
Skin burn	1	1
Bile duct stenosis	1	0
Gastric hemorrhage	1	0
Abdominal infection	0	1
Small intestinal obstruction	0	1

Abbreviations: TACE, transcatheter arterial chemoembolization; RFA, radiofrequency ablation.

© 2021 Zhang YJ et al. *JAMA Network Open*.

eFigure. Overall survival curves for patients with recurrence in the transcatheter arterial chemoembolization (TACE) plus radiofrequency ablation (RFA) and RFA groups.

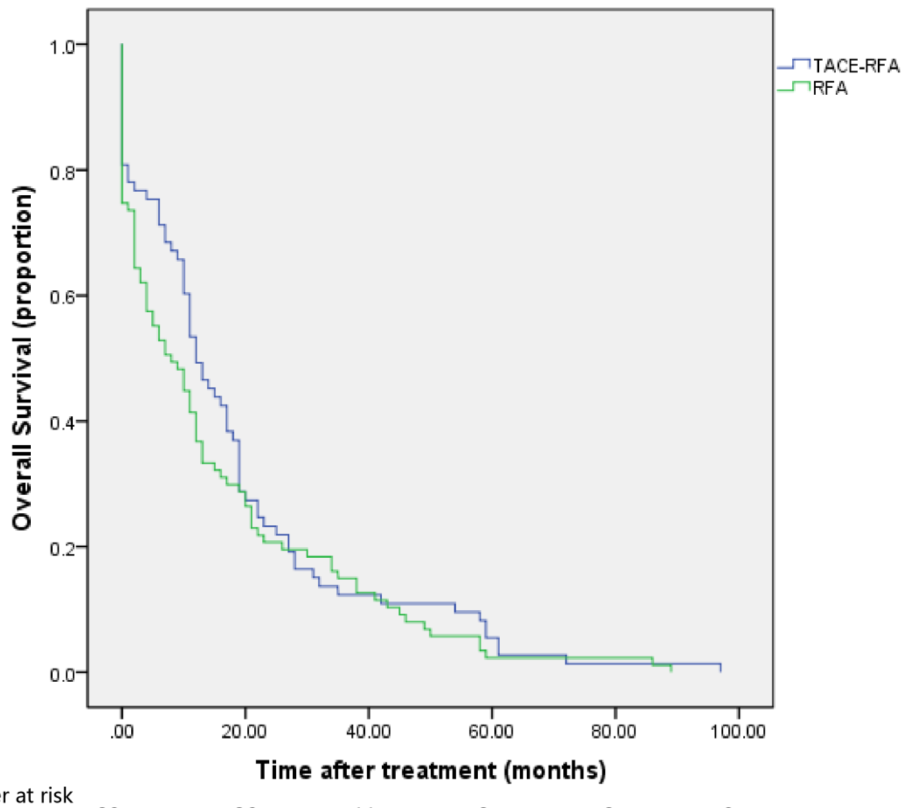

Number at risk						
TACE-RFA	80	26	11	3	2	0
RFA	87	24	10	3	2	0

© 2021 Zhang YJ et al. *JAMA Network Open.*
